# Soil Water Content Assessment: Critical Issues Concerning the Operational Application of the Triangle Method

**DOI:** 10.3390/s150306699

**Published:** 2015-03-19

**Authors:** Antonino Maltese, Fulvio Capodici, Giuseppe Ciraolo, Goffredo La Loggia

**Affiliations:** Department of Civil, Environmental, Aerospace, Materials Engineering, Università degli Studi di Palermo, Viale delle Scienze Ed. 8, 90128 Palermo, Italy; E-Mails: fulvio.capodici@unipa.it (F.C.); giuseppe.ciraolo@unipa.it (G.C.); goffredo.laloggia@unipa.it (G.L.L.)

**Keywords:** soil moisture, airborne remote sensing, triangle method

## Abstract

Knowledge of soil water content plays a key role in water management efforts to improve irrigation efficiency. Among the indirect estimation methods of soil water content via Earth Observation data is the triangle method, used to analyze optical and thermal features because these are primarily controlled by water content within the near-surface evaporation layer and root zone in bare and vegetated soils. Although the soil-vegetation-atmosphere transfer theory describes the ongoing processes, theoretical models reveal limits for operational use. When applying simplified empirical formulations, meteorological forcing could be replaced with alternative variables when the above-canopy temperature is unknown, to mitigate the effects of calibration inaccuracies or to account for the temporal admittance of the soil. However, if applied over a limited area, a characterization of both dry and wet edges could not be properly achieved; thus, a multi-temporal analysis can be exploited to include outer extremes in soil water content. A diachronic empirical approach introduces the need to assume a constancy of other meteorological forcing variables that control thermal features. Airborne images were acquired on a Sicilian vineyard during most of an entire irrigation period (fruit-set to ripening stages, vintage 2008), during which *in situ* soil water content was measured to set up the triangle method. Within this framework, we tested the triangle method by employing alternative thermal forcing. The results were inaccurate when air temperature at airborne acquisition was employed. Sonic and aerodynamic air temperatures confirmed and partially explained the limits of simultaneous meteorological forcing, and the use of proxy variables improved model accuracy. The analysis indicates that high spatial resolution does not necessarily imply higher accuracies.

## 1. Introduction

Detailed knowledge of the surface soil water content of the soil-vegetation system is crucial to the operational modeling of drought monitoring [1] and irrigation management [2]. During water shortages in semi-arid regions, the first response is to reduce the agricultural water allotments. To avoid reduction in crop productivity, accurate estimates of plant water requirements and plant water availability are needed to avoid wasting resources. In addition, in vineyard management, grape yield and quality depend on the soil water content in the root zone. Water stress is necessary to regulate vegetative and berry growth and quality; however, excessive stress can result in severe damage to fruit development, thus affecting metabolism and production.

At the basin scale, long-term rainfall reduction observed in several regions, including the Mediterranean [3], concurrent with extreme rainfall events can causes increased flash flooding. Even in this framework, a precise estimate of the antecedent surface soil water content, θ, improves flood prediction and the reliability of warning systems because soil water content controls the precipitation partition between infiltration and runoff [4]. In addition, the runoff has a significant impact on soil loss [5].

Many methodologies have been developed using remotely sensed data acquired in distinct regions of the electromagnetic spectrum, including passive and active microwave methods and the triangle and thermal inertia methods used with shortwave and longwave data. Each method has its own advantages and disadvantages. Chaouch *et al.* [6] inverted a microwave transfer model to retrieve θ from passive microwave data at low spatial resolution (≈50 km), which is unsuitable for small areas. Active remote sensing methods are based on backscattered signals in microwaves (RADAR). Their sensitivity depends on two main groups: sensor parameters, including frequency, incidence angle, and polarization; and soil parameters, which depend on geometric (soil roughness and vegetation biomass, height, fractional cover, structure) and dielectric (texture, organic content, salinity and water content) properties. To estimate θ from radar backscattering, empirical, semi-empirical, and theoretical models have been implemented. Empirical models (e.g., [7]) obtain accurate results under the setup conditions; theoretical models (e.g., [8]) are implemented under a theoretical basis to predict backscattering in response to sensor and soil parameters. Among the semi-empirical models, Capodici *et al.* [9] recently set up a coupled algorithm for θ estimation using co- and cross-polarized imagery, obtaining satisfactory assessments.

Within the field of passive remote sensing, many studies report the use of thermal and visible/near infrared (VIS/NIR) bands to determine soil surface water content. Because such images do not directly measure soil water, a number of methods have been developed to exploit relationships among θ and surface radiometric properties that can be more readily quantified.

Two such methods are the “thermal inertia” and “triangle” methods. The thermal inertia method (e.g., [10,11,12]) determines θ using a combination of thermal and visible images. Because the ability of a material to accumulate heat and release it within a defined time interval strongly depends on the water content of the material itself, θ can be indirectly inferred by measuring the variation of the body temperature over time. In particular, the short wave albedo, compared to the day-night temperature difference, can be used to estimate the soil surface water content because the amplitude of the temperature variation is a direct consequence of the amount of energy reaching the soil in the shortwave and of the soil properties, including bulk density, thermal conductivity, and heat capacity. For bare ground, soil thermal properties strongly depend on water content and soil density. Thermal inertia is therefore higher for wetter soils because water in the soil pores absorbs heat, and thus soil temperature changes more slowly than in dry soils.

Because the variation in surface temperature depends on the surface water content, the triangle method exploits this relationship [13] through evaporation over bare soil and transpiration over dense vegetation. Vegetation cover determines applicability of these methods: (i) the triangle method depends on the temperature difference between canopy and air, a relationship based on canopy resistance that varies with soil water availability; thus, the method is more suitable for vegetated than bare soils; (ii) in contrast, operational thermal inertia is based on the hypothesis that some fluxes are linearly related to surface temperature; thus, the method is more suitable for bare soil than vegetated areas.

Our research focuses on a method based on the feature space defined by optical and thermal data. In particular we discuss advantages and limitations of the triangle method and a simplified version of the thermal inertia method. According to Petropoulos *et al.* [14], the wide base of the triangular envelope depicts the relatively higher sensitivity of the bare soil temperature to its water content changes compared to the much lower sensitivity of progressively denser vegetation, which is highlighted by the triangle’s much narrower vertex.

We implemented a model based on the empirical approach of the triangle method [15,16], named for the triangle enveloping the typical trapezoidal shape of the scatterplot of the land surface temperature (LST) *vs.* a vegetation index (VI), such as the Normalized Difference Vegetation Index (NDVI). The minor trapezoid base corresponds to densely vegetated areas and highlights the low temperature variability caused by the thermoregulation mechanism of the plants, which are able to take water (if available) from soil layers not directly linked to soil surface evaporation. In contrast, the major base of the trapezoid corresponds to low or absent vegetation coverage and confirms the strong relationship between surface θ and surface temperature of bare soils.

Each interval of vegetation coverage can be classified by the direct correspondence between the “warm edge” characterized by dry conditions and thus also referred to as “dry edge”, and the “cold edge” characterized by wet conditions and alternatively named “wet edge”. The soil water content index can then be defined as a function of the relative position to the triangle edges of a generic point of the scatterplot. One assumption is that the time series extent be sufficient to include all possible soil water content conditions (from residual to saturation) and soil vegetation coverage to assess θ based on the hypotheses of linear variation of isopleths from the wet to the dry edge.

## 2. Theoretical Background

The soil water content is evaluated using both VIs and surface temperatures via the triangle method. A fundamental assumption is that, given a large number of pixels describing a full range of soil surface water content and fractional vegetation cover, sharp boundaries in the data describe real physical limits: bare soil, pixels fully covered by vegetation, and lower and upper limits of the surface θ (completely dry and at field capacity) [15].

Under these hypotheses, Jackson *et al.* [17] showed that air temperature minus canopy temperature can be used as an index of crop water stress. Their results are based on the surface energy balance equation for a crop canopy, taking into account the net radiation and soil heat flux (*R_n_* and *G_0_*, respectively) and the sensible and latent heat fluxes (*H* and λ*E*, respectively). Under atmospheric neutral conditions, sensible and latent heat fluxes can be expressed as $H=\rho c_{p}\left(T_{c}-T_{a}\right)/r_{a}$ and $\lambda E=\rho c_{p}\left(e_{c}^{*}-e_{a}\right)/\gamma \left(r_{a}+r_{c}\right)$, where ρ is the air density, *c_p_* is the air heat capacity, *T_c_* and *T_a_* are the canopy and air temperatures, e_c_^*^ and *e_a_* are the saturated and vapor pressures of the air, γ is the psychrometric constant, and *r_a_* and *r_c_* are the aerodynamic and canopy resistances to water transport.

The temperature difference between canopy and above air can be written as [17]:

(1) $$T_{c}-T_{a}=\frac{r_{a}\left(R_{N}-G_{0}\right)}{\rho c_{p}}\cdot \frac{\gamma \left(1+\frac{r_{a}}{r_{c}}\right)}{\Delta +\gamma \left(1+\frac{r_{a}}{r_{c}}\right)}-\frac{e_{a}^{*}-e_{a}}{\Delta +\gamma \left(1+\frac{r_{a}}{r_{c}}\right)}$$

where Δ is the slope of the saturated vapor pressure *vs.* temperature relationship (e_c_^*^ − e_a_^*^)/(*T_c_* − *T_a_*), and e_a_^*^ − *e_a_* is the vapor pressure deficit. Theoretically, Equation (1) can be used to define two boundary limits for well-watered and dry crops.

For well-watered crops and full cover, the canopy resistance reaches potential value, *r_c_* = *r_cp_*, whereas in bare soil, *r_c_* is approximated as zero. The potential value of canopy resistance is obtained per unit of Leaf Area Index (LAI) from minimum stomata resistance: *r_cp_* = *r_Smin_*/LAI [18]. These resistances determine the theoretical lower limit (wet edge).

For no water availability and full vegetation cover, the canopy resistance rises to the stomata closure value *r_c_* = *r_cx_* = *r_Smax_*/LAI as a function of the leaf stomata closure value [18], whereas for dry bare soil *r_c_* is set to infinity, *r_c_* = ∞. These resistances determine the theoretical upper limit (dry edge).

Following Ortega-Farias *et al.* [19], *r_cp_* over vineyards could be set to 25 s∙m^−1^ (cultivar Savignon), although values ranged between 10 and 100 s∙m^−1^ [20]. By following Giordani *et al.* [21], *r_cx_* over vineyards associated with nearly complete stomata closure could be set to 2000 s∙m^−1^. Thus, defining the value that describes the actual behavior of the vegetation is difficult. Regarding LAI influence, if none of the controlling variables is limiting, *r_s_* could be roughly assumed to be 40 s∙m^−1^ [22].

Net radiation is obtained by summing shortwave and longwave net radiations: $R_{N}=\left(1-\alpha_{SW}\right)R_{SW}+\sigma \left(\varepsilon_{a}\varepsilon_{c}T_{a}^{4}-\varepsilon_{c}T_{c}^{4}\right)$. The equation is implicit for surface temperature and thus must be solved iteratively.

Canopy resistance (*r_c_*) can be expressed as a function of the bulk stomata resistance (*r_s_*) per unit of active (or green) LAI that actively contributes to the surface heat and vapor transfer:

(2) $$r_{c}=\frac{r_{s}}{LAI}$$

The bulk stomata resistance is crop-specific and differs among crop varieties (and crop management). Because it usually increases as the crop matures and ripens, it is difficult to accurately evaluate in operational applications.

Stomata resistance depends on several environmental variables, including photosyntetically active radiation (PAR); atmospheric water vapor deficit (AMD); atmospheric carbon dioxide concentration (CO_2_), air temperature (*T_a_*); and θ. Conventionally, stomata resistance (*r_s_*, defined as the inverse of conductance, *G* = *r_s_^−1^*), is parameterized as a function of reduction coefficients accounting for these actual environmental variables, based on their minimum values observed under optimal conditions: $r_{s}=r_{s\text{min}}/\left(F_{\text{PAR}}F_{\text{AMD}}F_{T_{a}}F_{CO_{2}}F_{\vartheta }\right)$.

Within the diachronic empirical approach, the mutually compensating effect of meteorological forcing (PAR, AMD, *T_a_* and CO_2_) on *r_s_* must be verified. This evaluation must occur at least at acquisition time, and preferably carried out with the same sun elevation angle (airborne platform) or at the same time of day (sun-synchronous satellites).

The empirical method bypasses efforts in assessing *r_c_* by adjusting dry and wet edges to the experimental scatterplot. This means that improvements can be achieved by considering a VI time series and the difference (Δ*T*) between LST and a reference temperature T^*^. We tested different T^*^ and propose a procedure to select the best model parameters.

## 3. Methods

The triangular shape [23] characterizing the scatterplot, relies on the assumption that the temperature of an elementary heterogeneous surface is given by the linear combination of the soil and vegetation radiative temperatures, and these are in linear relationship with the evaporation and transpiration processes, respectively.

**Figure 1:**
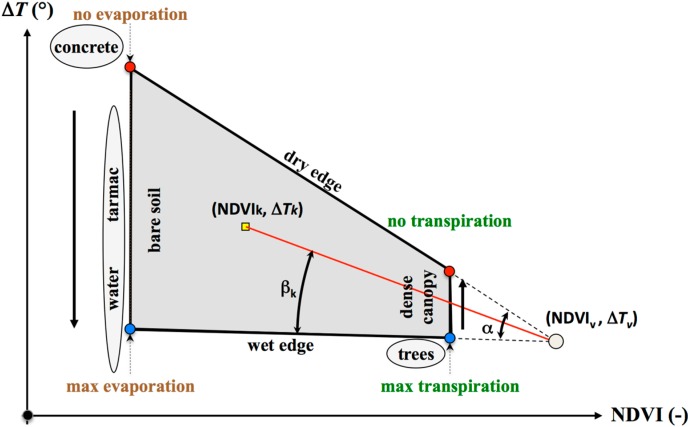
Scatterplot of NDVI *vs.* Δ*T* with superimposed dry and wet edges. Blue dots characterize maximum evaporation and transpiration, and minima are represented by red dots. The generic NDVI-Δ*T* pair is indicated by a K subscript; V subscript represents the triangle vertex.

Given the VI, the soil water content of a pixel is often assumed to be proportional to the ratio between its thermal difference with the dry edge compared to the total thermal excursion between dry and wet edges. Two straight lines identify dry and wet edges. Within this research, a θ index for the generic *k* element (NDVI*_k_*, Δ*T_k_*) is given by the ratio of two angles [24], β*_k_* and α. Angle β*_k_* is given by the wet edge and the straight line joining the generic pixel of the scatterplot and the triangle vertex. Angle α is between the dry and wet edges and is thus proportional to the whole variability (Figure 1). This index is then converted into absolute values using the residual (θ*_res_*) and saturation (θ*_sat_*) outer values. This index strongly depends on the positioning of the dry and wet edges, which are determined by means of linear regression of minima and maxima percentiles for a given NDVI class.

The residual and saturated θ values, θ*_res_* and θ*_sat_*, were determined by laboratory analyses on samples collected within the study area.

### 3.1. Edge Determination through a Diachronic High Spatial Resolution Dataset Acquired on a Small Area

**Figure 2:**
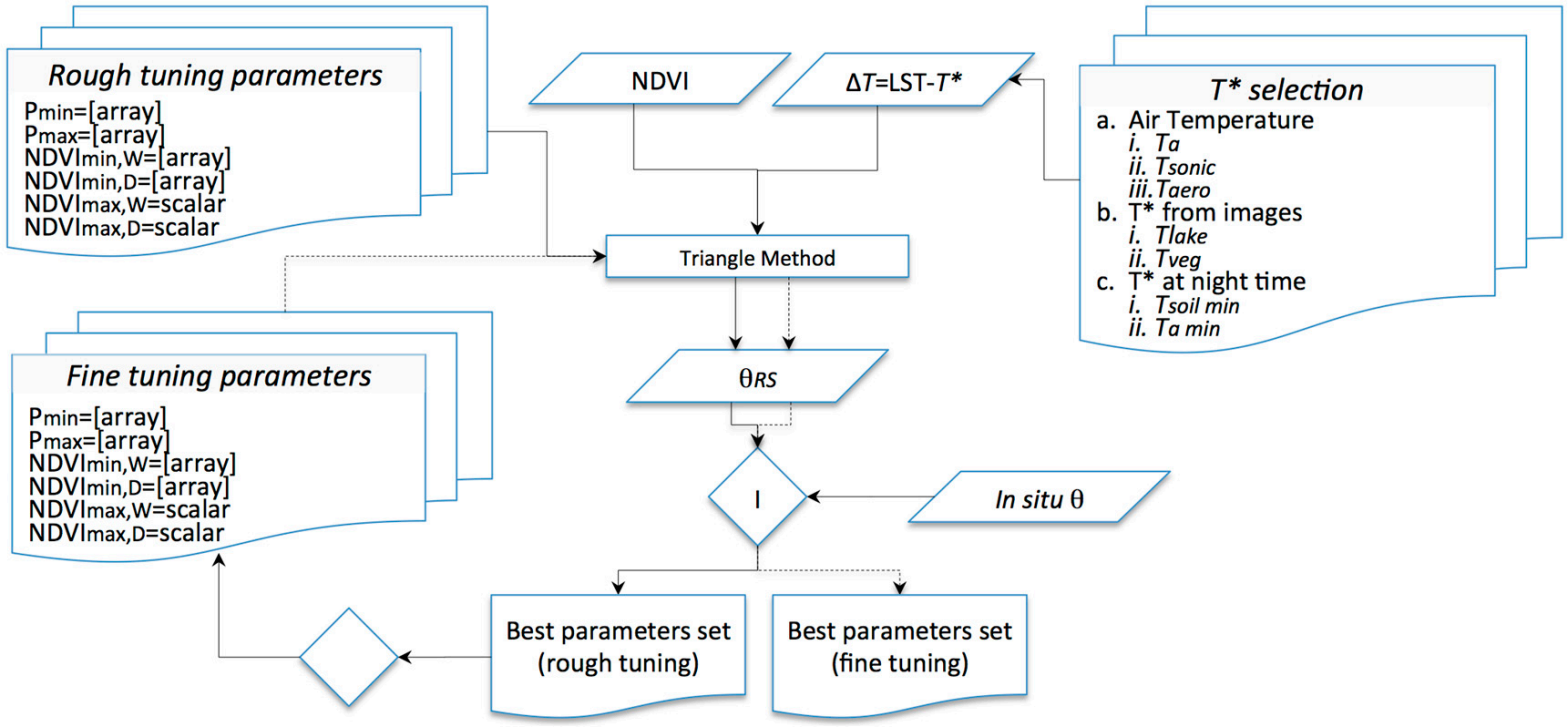
Flow diagram of the best fitting parameters procedure; dashed connectors indicate a fine-tuning procedure.

This analysis aimed to determine how an operational triangle method approach can be developed to assess θ over a small, vegetated area using a diachronic Earth Observation (EO) dataset of thermal and VIS/NIR images characterized by high spatial resolution. Once the method is applied on an empirical basis, wet and dry edges can be determined directly from the optical-thermal feature space by setting up few parameters (Figure 2, tuning parameters). Over small areas, a diachronic approach is valuable because time-series describe, for given vegetation cover, a wider range of variability of θ; thus, including all the NDVI-Δ*T* pairs within the scatterplot could be opportune (and in some cases needed).

The use of high spatial resolution does not necessarily imply best accuracies in θ retrieval, and thus the appropriate resolution of θ maps requires a further discussion. Wet and dry edges must be assessed at the finest spatial resolution (usually the resolution of thermal images). Spatial aggregation reduces both the range of variability and total number of pairs, threatening the characterization of θ minima and maxima values.

### 3.2. Parameter Tuning

Parameters were defined to optimize the match between remote sensing θ (θ*_Rs_*) and θ measured *in situ* (Figure 2). Parameters included minima and maxima percentiles (*P_min_* and *P_max_*) and minimum NDVI of the wet and dry edges (NDVI*_min,D_* and NDVI*_min,W_*); whereas the cluster shape allows fixing maximum NDVI (NDVI*_max,D_* and NDVI*_max,W_*) with confidence.

The wet and dry edges were determined by applying a linear regression to the minimum and maximum percentiles of NDVI classes; this choice gives a certain degree of freedom to the operator because it directly influences the final result. The experiment shows that percentiles cannot be set *a priori*, and thus, the percentiles that best fit the *in situ* data must be determined.

Increasing *P_min_* and decreasing *P_max_* bring the edges closer to the cluster. The choice of different percentiles determines the value of α (Figure 1), representing the range of variability of θ (Δθ). The more *P_min_* increases, the more β*_k_* decreases (Figure 1); consequently, θ rises. As a first attempt (rough tuning) the following percentiles were tested: *P_min_* = [2, 5, 10, 20, 30] and *P_max_* = [98, 95, 90, 80, 70]. Resulting maps represent θ driving evaporation and transpiration of the soil vegetation system; however, pixels characterizing other elements of the study area could still remain within the scatterplot. Those pixels could distort the expected trapezoidal shape of the cluster. Hence, it is possible, sometime necessary, to remove them. Although input images can be masked, both NDVI*_min,D_* and *NDVI_min,W_* have to be set to this aim. During the rough tuning, minimum NDVI arrays of NDVI*_min,W_* = NDVI*_min,D_* = [0.1, 0.2, 0.3, 0.4] were tested, and NDVI*_max,W_* and NDVI*_max,D_* were fixed to 0.90 and 0.99, respectively. Parameters led to 450 model runs for each T^*^.

### 3.3. Reference Temperature Tests

Although in principle *T_a_*, measured simultaneously with LST should be employed, a weak agreement with *in situ* θ suggests testing alternative T^*^ (Table 1). In particular, two tests were conducted to analyze (a) whether *T_a_* does or does not work ($T_{AIR}^{*}$ test); (b) if operational T^*^ directly retrieved from thermal images could be used ($T_{IMAGE}^{*}$ test); and (c) if including thermal admittance could lead to more accurate results ($T_{ADMITTANCE}^{*}$ test).

**Table 1:** T^*^ used within $T_{AIR}^{\text{*}}$ $T_{IMAGE}^{\text{*}}$ and $T_{ADMITTANCE}^{*}$ tests.

$T_{AIR}^{*}$ Test	$T_{IMAGE}^{*}$ Test	$T_{ADMITTANCE}^{*}$ Test
T_a_	T_lake_	T_soil_min_
T_sonic_	T_veg_	T_a_min_
T_aero_	-	-

The $T_{AIR}^{*}$ test includes:

(i) *T_a_* measured by a thermoigrometer installed 2.75 m above ground level (a.g.l.), ≈1 m above vegetation top;
(ii) the sonic air temperature (*T_sonic_*), retrieved by sound velocity measured by a 3D sonic anemometer installed ≈3 m a.g.l.; its value closely approximates *T_a_*. This test verifies if the weak results obtaining using *T_a_* are eventually due to thermoigrometer malfunctioning;
(iii) the aerodynamic temperature (*T_aero_*) retrieved by Two-Source Energy Balance model based on intercalibration (TSEB-IC [25]). Although *T_aero_* is not suitable for an operational use, it determines turbulent fluxes that underlie the theoretical basis of the method, thus identifying the limits of *T_a_* in describing the fluxes.

The $T_{IMAGE}^{*}$ test assesses the operational use of temperatures of particular targets, eventually included within the scene:

(i) the water surface temperature of irrigation ponds (*T_lake_*);
(ii) the temperature of dense-well watered vegetation (*T_veg_*).

The $T_{ADMITTANCE}^{*}$ test verifies if accuracy is improved when thermal admittance is accounted for; to this aim, two temperatures were tested:

(i) the minimum soil surface radiometric temperature (*T_soil_min_*);
(ii) the minimum air temperature (*T_a_min_*), which is easier to obtain because it is usually measured by standard meteorological stations.

### 3.4. Parameter Configuration and Reference Temperature Selection

The choice of the reference temperature and parameter configuration characterizes θ*_Rs_* coherently to the measured θ. The choice is based on the determination coefficient (*r^2^*), Student test value (*T*), Fischer test value (*F*), and the difference between maximum and minimum percentiles used to define the dry and wet edges (Δ*P*). Parameters must be configured to maximize *r^2^*, *T*, *F*, and Δ*P*; thus, a synthetic parameter configuration maximizing the product, I = *r^2^·T·F*·Δ*P*, was chosen. The mean absolute error (MAE) is qualitatively verified to assume a low value. The slope and intercept (*m* and *q*, respectively) between estimated and measured data are qualitatively verified to approach the unity (*m*), and the null value (*q*). After the first approximation of the parameters, fine-tuning yields the optimal configuration.

### 3.5. Optimal Spatial Aggregation

**Figure 3:**
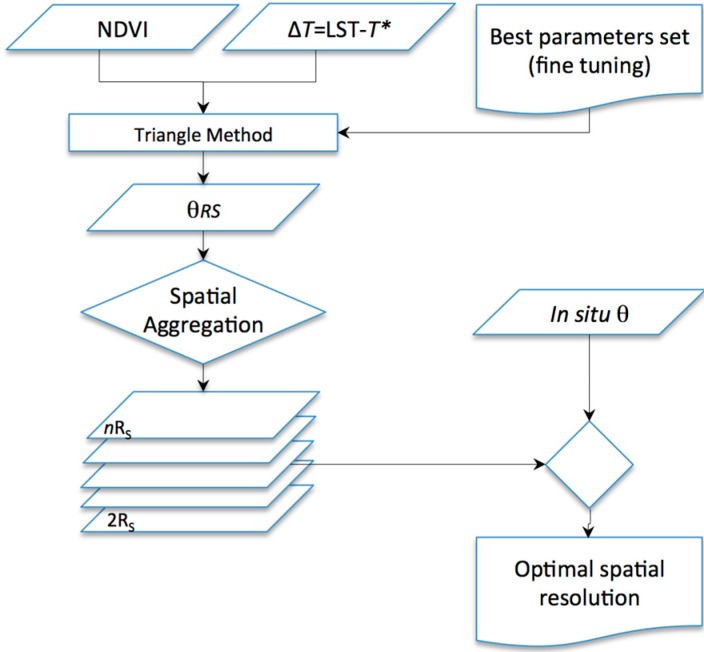
Flow diagram for the optimal spatial resolution determination.

Parameters are configured at the spatial resolution of the thermal acquisition using the whole diachronic dataset; the whole dataset eventually accounts for a sample size large enough to characterize dry and wet edges for the whole range of the vegetation index (outer θ conditions). Thus, the output *R_S_* of θ is originally the one of thermal acquisition. Nevertheless, not necessarily the better *R_S_* (more detailed) implies the better accuracy of θ; this is could be due to several factors including the spatial variability of the soil vegetation system, the bidirectional reflectance distribution function (BRDF) of both thermal and optical (VIS/NIR) bands, and the row orientation. Thus, the spatial aggregation of θ maps could increase their accuracy. Different aggregation scales were tested (2, 3, 4, 5 and 6 times *R_S_*), and the aggregation *R_S_* representing the best agreement with the *in situ* data was selected by maximizing *r^2^*, minimizing *q* and MAE, and approximating *m* to unity (Figure 3).

## 4. Study Area

The study area includes the experimental fields of the “Tenute Rapitalà” farm within the Camporeale district (Sicily), a specific zone that grants the Alcamo Denomination of Controlled Origin (DOC) status. The fields are characterized by an average slope of 10%, mainly S–SW aspect (<100°), and elevations ranging from 290 to 320 m a.s.l.

**Figure 4:**
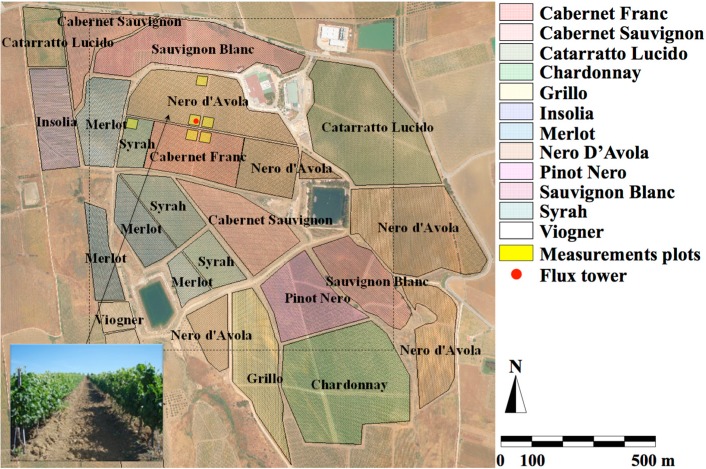
The study area included: vine cultivars (reported on the right side legend); the flux tower position (red dot), *in situ* measurements plots (highlighted in yellow); airborne image footprint (dashed black rectangle); canopy management (vertical trellis system) (lower-left box).

The soil texture (USDA) was classified as loam (20% clay, 29% silt) to silty clay loam (37% clay, 45% silt). The organic content ranged between 1.4% and 1.7%. The residual and saturation volumetric water contents (θ*_R_* and θ*_S_*) were 0.040 and 0.453 m^3^∙m^−3^, respectively, whereas volumetric water content at field capacity (θ*_FC_*) was 0.239 m^3^∙m^−3^. According to Centeno *et al.* [26], permanent wilting point (θ*_WP_*) was assumed to be 0.096 m^3^∙m^−3^ (cultivar “Tempranillo”, sandy loam soil).

The experimental field included four vineyards separated by cross-shaped section-breakers. The vineyards were cultivated adopting a vertical trellis system (Figure 4, lower-left box), with the cultivar “Nero d’Avola” at NW and NE; the SE field was partly cultivated with “Cabernet Franc” cultivar and partly with “Nero d’Avola”, and finally the SW field was partly cultivated with “Syrah” cultivar and partly with “Nero d’Avola” (Figure 4). Other cultivars included “Insolia”, “Cabernet Sauvignon”, “Sauvignon Blanc”, “Merlot”, “Grillo”, “Catarratto Lucido” and “Viogner”. Plant spacing was 240 cm between rows and 95 cm between the plants within the rows. Plant density was 4386 plants per ha; rows were oriented N–NE to S–SW.

## 5. Data

Remote sensing images in the short and long waves were acquired by an airborne platform, SKY ARROW 650 TC/TCNS, at a height of ≈1000 m a.g.l. The VIS and NIR images (three bands) were acquired by means of a multispectral MS4100 camera (Duncantech, Auburn, CA, USA, 767–832, 650–690, and 530–570 nm), and the thermal infrared band (TIR) images were acquired by means of a SC500/A40M camera (Flir, Wilsonville, OR, USA, 7.5–13 μm). The nominal pixel resolution was approximately 0.6 m for the VIS/NIR bands and 1.7 m for the TIR channel. The VIS/NIR images were aggregated at a coarser spatial resolution. Details about the field campaign and remote sensing data can be found in Maltese *et al.* [24]. The installed micro-meteorological station is described in [27].

Volumetric water content was measured with a CS616 Water Content Reflectometer (Campbell Scientific, Inc., Logan, UT, USA). The probe consisted of two 30 cm long stainless steel rods introduced vertically in the soil between 10 and 40 cm below ground level. An ARG100 Tipping Bucket Raingauge (Campbell Scientific, Inc.) installed 1 m above the vegetation top measured rainfall, tipping once for each 0.2 mm of rain. LAI was measured using an optoelectronics instrument (LAI-2000 Plant Canopy Analyzer, by LI-COR^®^, Lincoln, NE, USA). Radiometric temperature of the bare soil was measured by an IRTS-P Precision InfraRed Temperature Sensor pyranometer (Campbell Scientific, Inc.).

Vineyards were characterized by low active LAI in the first ten days of June (≈1.2 m^2^∙m^−2^, the average value at pixel scale during the first two acquisitions). LAI increased up to ≈1.7 m^2^∙m^−2^ in the last ten days of July (third image acquisition) and decreased again to a minimum of ≈0.95 m^2^∙m^−2^ at the beginning of September (last acquisition).

Simultaneous to the image acquisitions, *in situ* measurements were carried out, including θ, measured using both the traditional thermo-gravimetric method and a handheld TDR (FieldScout TDR300, Spectrum Technologies Inc., Aurora, IL, USA). During each airborne acquisition date (11 June, 3 and 22 July, and 3 September 2008 corresponding to 163, 185, 204, 247 days of year, DOYs, respectively) 36 TDR measurements of bulk dielectric dielectric permittivity (ε) of the soil matrix were performed for each of the 6 selected plots (Figure 4): three (A, D and F) within the Nero D’Avola cultivar; two within a Cabernet Franc (C and D), and the last (E) in the Syrah plot. Half of these measurements were acquired in the upper soil layer (0–20 cm) and the remaining half in the upper part of the root zone (~25–35 cm), totaling about 1100 observations. Soil water content measured in the upper part of the root zone was compared with the remote sensing assessment. Additionally, soil samples were collected to apply the thermo-gravimetric method and to assess the bulk density, needed to convert reflectometric measurements into θ. The Soil Hydrology Laboratory of the Department of Engineering and Agro-Forest Technology (ITAF) of the University of Palermo performed the soil characterization (including grain size curve, residual and saturation θ, organic content, *etc.*).

The short wave images were calibrated to spectral reflectance and corrected for atmospheric influence, applying the empirical line method [28] requiring for ground reflectance measurements over targets taken simultaneously with the images. The thermal images were converted into surface radiometric temperatures by means of a linear regression with *in situ* temperature measurements using the emissivity maps retrieved as a function of the NDVI, as proposed by Sobrino *et al.* [29]. To do this, a number of spectroradiometric and radiometric measurements were performed on artificial surfaces (black and white panels) and on natural surfaces with homogeneous radiometric characteristics at the spatial acquisition scale (bare soils, roads, small irrigation reservoirs).

The vineyards were irrigated using a controlled water deficit technique, a drop irrigation system able to supply 4 L·h^−1^ to each plant. The farm irrigation strategy included nine drop irrigations ranging between 4 and 7 h in duration each from 28 May to 18 August. Watering was provided twice between the end of May and June and 4 times in the first twenty days of July during flowering, fruit setting, and veraison. Water management during this period has a direct effect on the subsequent berry average weight, and irrigation between the end of July and August aims to optimize growth as well as berry sugar and acidity contents.

Rainfall events during the study period were few and characterized by low accumulations. Values ranged between 6 and 8 mm in the first 4 events during 10–28 May, and the subsequent 3 months were dry until 14 September, when a significant rainfall occurred (196 mm).

During this research, the θ extremes chosen to characterize the dry and wet edges were 0.040 m^3^∙m^−3^ (θ_R_) and 0.453 m^3^∙m^−3^ (θ_S_), determined by *in situ* soil sampling and laboratory measurements.

A micro-meteorological station measured several variables, including PAR, *T_air_*, CO_2_, and θ*_air_*, during the whole period. Recorded data were used to evaluate the product of the stomata conductance reduction factors (*F_PAR_* *F_Ta_* $F_{CO_{2}}$ *F*_θ*air*_); their product was almost constant with time (variation coefficient ≈ 14%).

## 6. Results and Discussion

### 6.1. Parameter Tuning

**Figure 5:**
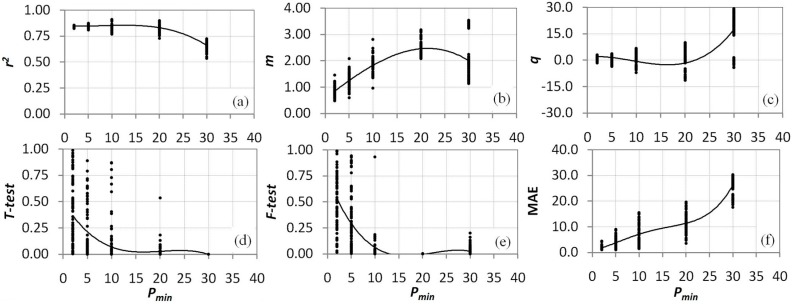
Variability with *P_min_* of some statistical parameters used to characterize *in situ* *vs.* remote sensing θ: (**a**) determination coefficient *r^2^*, top left panel; (**b**) slope *m*, upper central panel; (**c**) intercept *q*, upper right panel; (**d**) Student test *T-test*, lower left panel; (**e**) Fisher test *F-test*, lower central panel; (**f**) mean absolute error MAE, lower right panel; An interpolation line is reported in black.

As reported in the Methods section, the positioning of wet and dry edges was determined by applying a linear regression to the minimum and maximum Δ*T* percentiles of NDVI classes; thus, their positions depend on the choice of percentiles *P_min_* and *P_max_*. Statistics of *r^2^*, *T*-test, *F*-test, Δ*P*, and MAE (the case of T^*^ = *T_a_min_*, was chosen as an example) for varying *P_min_* *m*, and *q* (Figure 5) show clear uncertainties in the choice of a suitable *P_min_*. High ranges of variability of these indices confirm how the parameter settings affect the result. Thus, the use of a synthetic index (see index I, Section 3) could be valuable for the parameter selection. No noticeable behavior of these statistics has been found with *P_max_*.

For each T^*^, statistics characterizing *in situ* *vs.* remote sensing θ of the 450 model runs were analyzed. The process for selecting the best parameter set was explained in Methods. Both the best set of parameters and statistical indices characterizing *in situ* θ *vs.* θ*_Rs_* were synthesized (Table 2).

**Table 2:** Best-fit parameters for given temperature.

	*P_max_*	*P_min_*	Dry Edge		Wet Edge		*r^2^*	*m*	*q*	*T-test*	*F-test*	*MAE*	Δ*P*	*I*
			NDVI*_min_*	NDVI*_max_*	NDVI*_min_*	NDVI*_max_*								
*T_a_*	70	2	0.2	0.9	0.2	0.99	0.08	0.25	6.41	0.86	0.69	3.32	0.68	0.03
*T_sonic_*	70	5	0.2	0.9	0.2	0.99	0.29	0.52	5.00	0.54	0.91	2.76	0.65	0.09
*T_aereo_*	98	10	0.1	0.9	0.3	0.99	0.86	1.07	−1.41	0.58	0.57	1.39	0.88	0.25
*T_lake_*	98	10	0.1	0.9	0.2	0.99	0.88	1.10	−1.65	0.58	0.55	1.35	0.88	0.24
*T_veg_*	70	10	0.3	0.9	0.3	0.99	0.64	0.76	2.72	0.65	0.82	1.76	0.60	0.21
*T_soil_min_*	90	5	0.1	0.9	0.2	0.99	0.85	0.87	1.65	0.68	0.82	1.07	0.85	0.41
*T_a_min_*	95	5	0.1	0.9	0.3	0.99	0.87	0.90	1.58	0.58	0.89	1.09	0.90	0.40

Results of the $T_{AIR}^{*}$ test revealed that the best parameter setup using *T_a_* as reference temperature produces low accuracy maps (*I* ≈ 0.03), and *T_sonic_* revealed that these weak performances are not related to measurement inaccuracies or failures (providing a small increment of *I*, Δ*I* ≈ +0.06). *T_aero_* should drive actual *H* and, subsequently, λ*ET*; thus, better results are expected if *T_aero_* is used instead of *T_a_* because it determines turbulent fluxes. Results (Table 2) confirm these hypotheses (Δ*I* ≈ +0.22); thus, we conclude that *T_a_* is not suitable as reference temperature.

Results of the $T_{IMAGE}^{*}$ test showed that some reference temperatures directly retrievable on thermal images provided an accurate assessment of θ. In particular, the temperature of a small irrigation pond, *T_lake_*, achieved a similar level of agreement with *in situ* θ (Δ*I* ≈ +0.21) if compared to that obtained using *T_aereo_* (lower accuracy is obtained using *T_veg_*, Δ*I* ≈ +0.18). The $T_{IMAGE}^{*}$ test revealed that accurate results can be achieved without the use of ancillary meteorological data.

The $T_{ADMITTANCE}^{*}$ test confirmed that accuracy could be improved by accounting for thermal admittance. Indeed, agreement with *in situ* θ notably increased (Δ*I* ≈ +0.38). However, measures of surface soil temperature are not commonly available; thus, employing *T_soil_min_* has limited operational utility. From an operational standpoint we tested *T_a_min_* (easily available) as a surrogate of *T_soil_min_*. *T_a_min_* lead to results (Δ*I* ≈ +0.37) comparable to those achieved by *T_soil_min_*. The level of agreement with *in situ* θ was even higher if compared to that obtained using *T_aereo_*.

Because the best setup configuration using *T_a_min_* provided the higher Δ*I* during the rough tuning procedure, T^*^ = *T_a_min_* was chosen for a subsequent fine-tuning of parameters to optimize the parameter setup (Table 3). Results revealed that all statistical indices are improved, in particular Δ*I* remarkably increased up to ≈+0.70.

**Table 3:** *T_a_min_* parameters fine-tuning.

	*P_max_*	*P_min_*	Dry Edge		Wet Edge		*r^2^*	*m*	*q*	*T-test*	*F-test*	*MAE*	*ΔP*	*I*
			NDVI*_min_*	NDVI*_max_*	NDVI*_min_*	NDVI*_max_*								
*T_a_min_*	97	7	0.1	0.9	0.3	0.99	0.89	0.96	0.46	0.95	0.96	0.83	0.90	0.73

**Figure 6:**
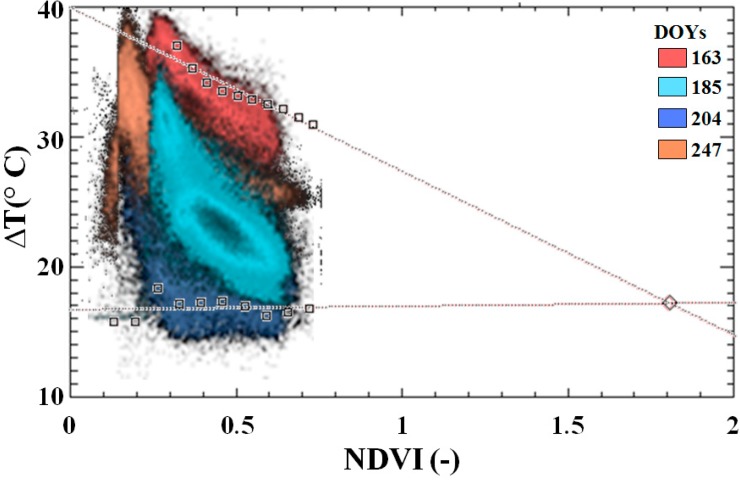
Scatterplot of NDVI *vs.* Δ*T*. Over imposed dry (*P_max_* = 97) and wet edges (*P_min_* = 7). Pixels from different images are represented with colours ranging from red to blue, to indicate increasing average θ.

A scatterplot of NDVI *vs.* Δ*T* pairs for the whole diachronic dataset is plotted in Figure 6 (red, orange, cyan and blue color ramp indicates increasing θ). Individual clusters highlight that an EO acquired on a small area during a single DOY was not able to characterize both dry and wet edges, and a diachronic dataset is required.

Minima θ were reached on DOY 163 (pairs drawn in red) and determined the dry edge, whereas maxima θ were reached on DOY 204 (pairs represented in blue) and determined the wet edge.

### 6.2. Spatial Resolution Analysis

**Figure 7:**
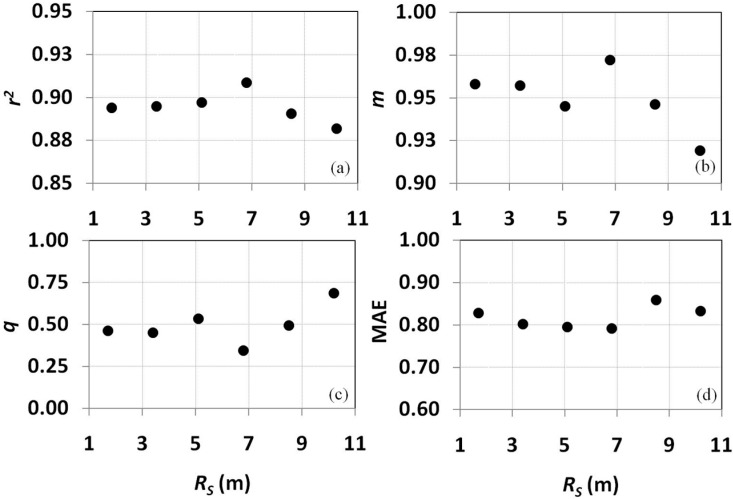
Variability of some statistical parameters used to characterize *in situ* *vs.* remote sensing θ at increasing θ*_Rs_* aggregation scale: (**a**) *r^2^*, upper left; (**b**) *m*, upper right panel; (**c**) intercept *q*, lower left panel; (**d**) MAE, lower right panel.

As reported in Methods, higher *R_S_* do not necessarily lead to higher accuracy of θ maps; thus, different aggregation scales were tested (Figure 7).

Six times *R_S_*, 6.8 m (approximately four times the distance between rows, 7.2 m), improved the retrieval accuracy; *r^2^* increased from ≈0.89 to ≈0.91; *m* approached the unity, from ≈0.96 to ≈0.97; and *q* and MAE were minimized, from ≈0.43 and ≈0.83 to ≈0.35 and ≈0.79, respectively. Results are significant mainly because comparable or even better accuracy can be achieved by acquiring images at lower *R_S_*, thus reducing the imaging cost.

### 6.3. Soil Water Content Spatial Distribution

**Figure 8:**
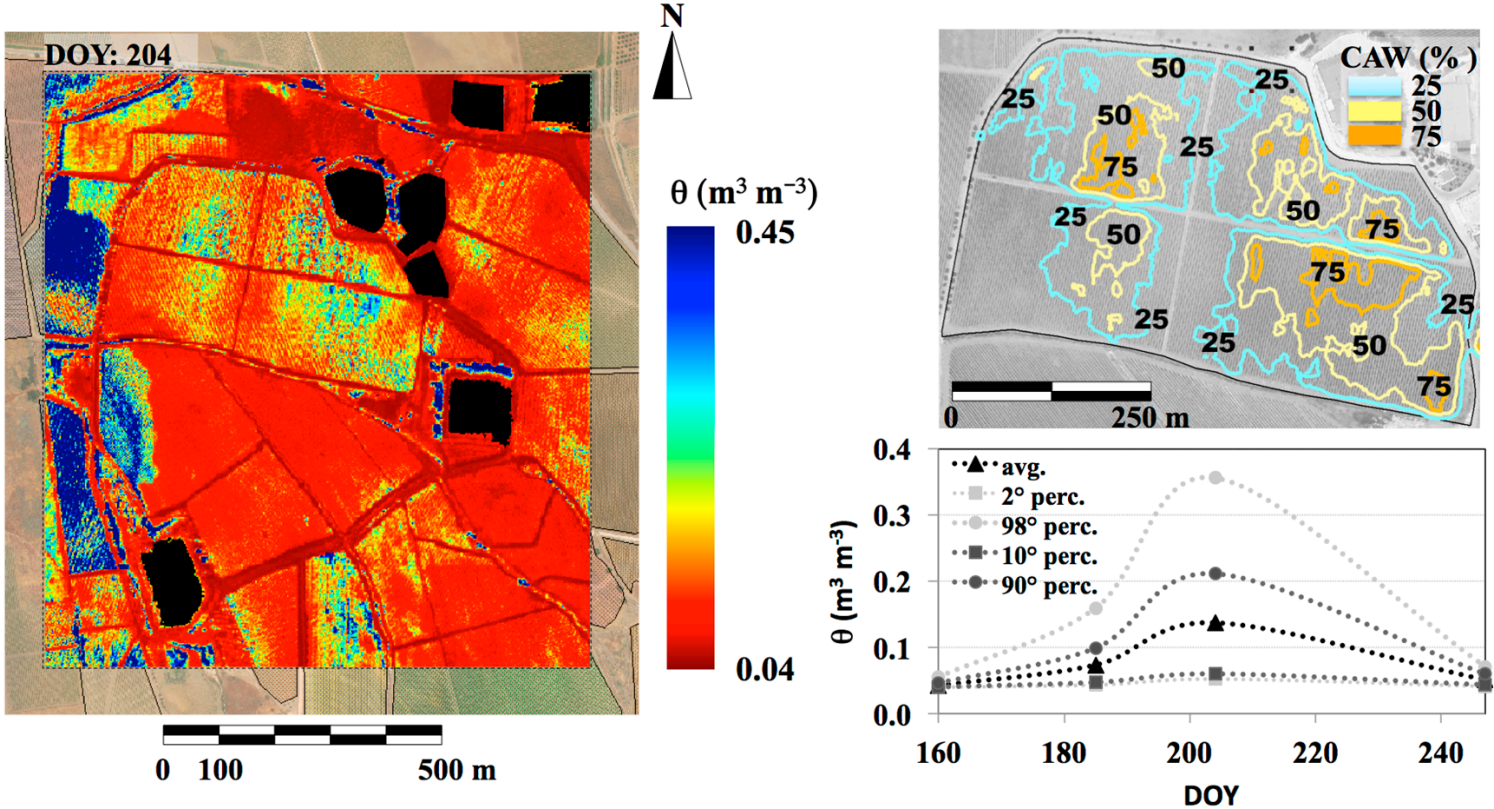
Spatial distribution of θ for the DOY exhibiting the higher spatial variability (DOY 204, left panel) and current percentage of available water to the plant roots (upper-right panel); θ statistics (2nd, 10th, 90th, 98th percentiles and average spatial values) for the whole time series (lower-right panel).

The θ map characterizing DOY 204 (Figure 8, left panel; lakes and buildings are masked out) was chosen because it exhibits the higher spatial variability. The temporal variability (Figure 8, right panel) shows that θ approaches the dry edge on the whole area at the beginning of irrigation period (DOY 163); on DOY 185, average θ increases, and its distribution shows a statistical tendency toward wet pixels; the skewness is confirmed on DOY 204 when θ values are maximized. Finally, at the end of the irrigation period (DOY 247), soil dries and θ average value and distribution are similar to the beginning of the irrigation period.

In irrigation practices, only a percentage of available water capacity (AWC) is depleted because plants start to experience water stress even before soil water reaches θ*_wp_*. Therefore, a maximum allowed depletion (MAD, %) of the AWC must be specified. For example, MAD values of 50%–55% were used by Ortega-Farias and Acevedo [30] to schedule the irrigation of a Cabernet Sauvignon vineyard located in Chile. The actual percentage of available water (CAW) to the plant roots (CAW = θ − θ*_WP_*/θ*_FC_* − θ*_WP_*) at the acquisition time at *R_S_* = 6.8 m is plotted in Figure 8 (upper right panel). The isopleths allow identifying areas above a chosen MAD.

## 7. Conclusions and Outlook

This research highlights the limits of the triangle method if applied to small areas with high spatial resolution images on a strict theoretical basis. Additional efforts are required to parameterize the theoretical method. The empirical method bypasses these efforts by setting up some parameters and adjusting the outer edges bounding the feature space defined by optical and thermal images. An operational procedure was proposed that exploited a diachronic approach to reduce operator influence on setting up model parameters. Results confirmed that the use of a diachronic approach includes a wider range of variability of soil water content (θ) given the vegetation cover, thus making the method applicable on small areas using high spatial resolution data.

Different alternative reference temperatures were tested, and some achieved high θ accuracy. Results revealed that if measures of simultaneous air temperature (*T_a_*) are used as reference temperature, weak agreement with *in situ* θ is achieved (*r^2^* ~ 0.1, MAE ~ 3.3). The unsuitability of *T_a_* was confirmed by the weak results achieved also using the sonic temperature (*T_sonic_*) (*r^2^* ~ 0.3, MAE ~ 2.7) and by the accurate results obtained using the aerodynamic temperature (*T_aero_*) (*r^2^* ~ 0.9, MAE ~ 1.4). The retrieval of reference temperatures directly from thermal images (if suitable targets are available) promises advantages. The water surface temperature of an irrigation pond (*T_lake_*) and the temperature of a well-watered dense vegetation (*T_veg_*) were tested. According to the *in situ* θ, *T_lake_* was better suited as a reference temperature (*r^2^* ~ 0.9, MAE ~ 1.3) than *T_veg_* (*r^2^* ~ 0.6, MAE ~ 1.8).

The $T_{ADMITTANCE}^{*}$ test closely agreed with *in situ* θ, confirming that minima temperatures of soil (*T_soil_min_*) allows accounting for the soil admittance (*r^2^* ~ 0.85, MAE ~ 1.1); if the *T_soil_min_* is unknown, the use of minimum air temperature (*T_a_min_*) as surrogate also yields accurate assessments (*r^2^* ~ 0.9, MAE ~ 1.1). In addition, these data are available by standard meteorological stations.

A final test was employed to verify if the higher spatial resolution implies more accurate results compared with *in situ* observations. This test, performed by aggregating the remotely assessed θ on the best T^*^ and model parameter set, confirmed that the optimum spatial resolution (*r^2^* ~ 0.9, MAE ~ 0.8 at *R_S_* ~ 7m) is a multiple of the spatial land fragmentation of the observed soil-vegetation system (~three times the distance between rows, in this case).

There are concerns about the assumption of linear behavior of the isopleths within the temperature-vegetation index space. Stisen *et al.* [31] suggested a nonlinear interpretation of the surface temperature-vegetation index domain. Krapez *et al.* [32] simulated the isopleths of the root zone between residual and saturation values within a T-NDVI space using the SEtHyS model [33]. Simulated isopleths were nonlinear, showing curvatures for low and high vegetation covers. Curvature for low vegetation cover may originate from aerodynamic parameterization for bare or scarcely vegetated soils; nonlinear behavior for dense vegetation is reduced for high root zone water content.

Future research must take into account the nonlinear behavior of the isopleths within the temperature-vegetation index space and the different sensitivities of bare soil temperature to θ changes if compared with the vegetated surfaces.

## Abbreviations

**Symbol**: **Meaning**
*c_p_*: Air heat capacity
*e_c_^*^* and *e_a_*: Saturated and vapor pressures of the air, respectively (resp.)
*k*: Generic element of the scatterplot
*m* and *q*: Slope and intercept between estimated and measured data, resp.
*r^2^*: determination coefficient
*r_a_* and *r_c_*: Aerodynamic and canopy resistances to water transport
*r_c_* and *r_s_*: Canopy and stomata resistances, resp.
*r_cx_* and *r_cp_*: Stomata and potential closure resistances, resp.
*r_Smin_* and *r_Smax_*: Minimum and maximum stomata resistances, resp.
*F*: Fischer test value
*F_AMD_*, *F_CO2_*, *F_PAR_*, *F_Ta_*, *F*_θ_: Reduction coefficients for AMD, CO_2_, PAR, *T_a_* and θ, resp.
*G*: Stomata conductance
*G_0_*: Soil heat flux
*I*: Synthetic parameter configuration maximizing the product between *r^2^*, *T*, *F* and Δ*P*
*H*: Sensible heat flux
NDVI*_k_*: NDVI of the k element
NDVI*_min_* and NDVI*_max_*: Minimum and maximum NDVI, resp.
NDVI*_min,D_* and NDVI*_min,W_*: Minimum NDVI of the wet and dry edges, resp.
NDVI*_max,D_* and NDVI*_max,W_*: Maximum NDVI of the wet and dry edges, resp.
P_min_ and P_max_: Minima and maxima percentiles, resp.
*R_n_*: Net radiation
*T*: Student test value
*T_c_* and *T_a_*: Canopy and air temperatures, resp.
*T_a_min_*: Minimum air temperature
*T_aero_*: Aerodynamic temperature
*T_lake_*: Water surface temperature of irrigation ponds
*T_veg_*: Well watered dense vegetation temperature
*T_soil_min_*: Minimum surface soil radiometric temperature
*T_sonic_*: Sonic temperature
*T^*^*: Reference temperature
v: Subscript representing the triangle vertex
α: Angle between dry and wet edges
α*_SW_*: Shortwave albedo
β*_k_*: Angle individuated by the generic pixel
λ*E*: Latent heat flux
γ: Psychrometric constant
θ: Soil water content available in the root zone
θ*_cr_* and θ*_w_*: Critical θ and θ at the permanent wilting point, resp.
θ*_res_* and θ*_sat_*: Residual and saturation θ, resp.
θ*_Rs_*: Remote sensing derived θ
ρ: Air density
Δ*P*: Difference between maximum and minimum percentiles defining dry and wet edges
Δ: Slope of the saturated vapor pressure
Δ*I*: Variation of *I* resulting from using *T^*^* compared to *T_a_*.
Δθ: Range of variability of θ
Δ*T*: Thermal difference between LST and *T^*^*
Δ*T_k_*: Δ*T* characterizing the *k*-element

## Acronyms

AMD: Atmospheric water vapor deficit
AWC: Available water capacity
BRDF: Bidirectional reflectance distribution functions
DOC: Denomination of Controlled Origin
DOY: Day of the year
CAW: Percentage of current available water
CO_2_: Atmospheric carbon dioxide concentration
EO: Earth Observation
ITAF: Department of Eng. and Agro-Forest Technology
LAI: Leaf Area Index
LST: Land surface temperature
MAD: Maximum allowed depletion
MAE: Mean Absolute Error
NDVI: Normalized Difference Vegetation Index
PAR: Photosyntetically active radiation
*R_S_*: Spatial resolution
S, SW, N, NW, NE: South, South West, North, North West, North East
TDR: Time-domain reflectometry (method) or Time-domain reflectometer (instrument)
$T_{ADMITTANCE}^{*}$: Test employing minimum soil and air temperatures
$T_{AIR}^{*}$: Test employing air, sonic and aerodynamic temperatures
$T_{IMAGE}^{*}$: Test employing water lake and well watered dense vegetation temperatures
TIR: Thermal Infrared
TSEB-IC: Two-Source Energy Balance model based on intercalibration
USDA: United States Department of Agriculture
VI: Vegetation index
VIS: Visible spectral bands
NIR: Near Infrared spectral bands
